# Therapeutic Effects of Erythroid Differentiation Regulator 1 on Imiquimod-Induced Psoriasis-Like Skin Inflammation

**DOI:** 10.3390/ijms17020244

**Published:** 2016-02-17

**Authors:** Kyung Eun Kim, Younkyung Houh, Hyun Jeong Park, Daeho Cho

**Affiliations:** 1 Department of Life Systems, Sookmyung Women’s University, Chungpa-Dong 2-Ka, Yongsan-ku, Seoul 140-742, Korea; kyungeun@sookmyung.ac.kr (K.E.K.); hyk-602@daum.net (Y.H.); 2 Department of Dermatology, Yeouido St. Mary’s Hospital, The Catholic University of Korea, Seoul 150-713, Korea

**Keywords:** ERDR1, psoriasis, inflammatory skin diseases, Th17, CCR6, CCL20

## Abstract

Psoriasis is a common skin disease accompanied by chronic inflammation. In previous studies, erythroid differentiation regulator 1 (ERDR1) was shown to have a negative correlation with proinflammatory cytokine IL-18. However, the role of ERDR1 in the inflammatory skin disease psoriasis has not been evaluated. In this study, to investigate the role of ERDR1 in psoriasis, recombinant ERDR1 was injected intraperitoneally into a psoriasis mouse model. Recombinant ERDR1 (rERDR1) significantly alleviated the symptoms of psoriasis-like skin inflammation and reduced the mRNA of various psoriasis-related markers, including keratin 14, S100A8, and Th17-related cytokines IL-17 and IL-22, suggesting that rERDR1 exerts therapeutic effects on psoriasis via the regulation of Th17 functions. Additionally, the expression of CCL20, a well-known Th17 attracting chemokine, was determined. CCL20 expression significantly decreased in the rERDR1-injected group compared with the vehicle (PBS)-injected group. CCR6 expression in the psoriatic lesional skin was also decreased by rERDR1 administration, implying the inhibition of CCR6-expressing Th17 cell chemotaxis via the downregulation of CCL20. Taken together, this study provides the first evidence that ERDR1 may be a potential therapeutic target for psoriasis.

## 1. Introduction

Psoriasis is a common chronic skin disease that affects 2%–3% of the world’s population and manifests as red and white scaly plaques on the top layer of the skin. Although the pathogenesis of psoriasis is not fully understood, studies have demonstrated the involvement of immune-mediated cutaneous inflammation. Various stimuli such as stress, infection, and allergens stimulate keratinocytes to produce cytokines and chemokines in the dermal region, which trigger the extravasation of immune cells from blood vessels to lesional sites [1]. During the process, immune cells such as neutrophils, macrophages, dendritic cells, and type 1 T helper (Th1) cells are stimulated to create an inflammatory environment through interactions between the innate and adaptive immune systems [2]. As a result, resident skin cells, including keratinocytes, show abnormal hyper-proliferation and disturbed differentiation in the psoriatic lesional skin. Because the onset and maintenance of psoriasis is mediated by various inflammatory cytokines, therapeutic approaches focus on inhibition of certain inflammatory responses to improve disease symptoms [3,4].

The pathogenesis of psoriasis is a complex inflammatory process caused by the cytokine axis. Representatively, interleukin (IL)-17, IL-23, and tumor necrosis factor-α (TNF-α) initiate keratinocyte activation and hyper-proliferation, suggesting that these cytokines should commonly be considered markers of psoriasis [5,6]. In particular, recent studies have reported the critical roles of Th17 cells in psoriasis. Effector cytokines, such as IL-17 and IL-22, produced by Th17 cells, induce hyper-proliferation of keratinocytes, production of proinflammatory cytokines, angiogenesis, and inflammation as part of the pathogenesis of psoriasis [7]. Moreover, retinoid-related orphan receptor γt (RORγt), a specific transcription factor for Th17 cells, and Th17-inducing cytokines, such as IL-1β, IL-6, and IL-23, are significantly increased in psoriatic lesional skin [8]. Th17 cells express elevated levels of a specific cysteine–cysteine (CC) chemokine receptor CCR6 and migrate towards areas where the concentration of CCL20, the specific ligand for CCR6, is high. High-level expression of CCR6 and CCL20 in psoriatic lesional skin have been reported, and the abundant Th17 cells in lesional skin induce cellular changes, resulting in the recruitment of various immune cells, increased proinflammatory cytokines and antimicrobial peptides, and skin barrier dysfunctions [9,10,11]. Overall, these reports indicate a critical role for IL-17 in the pathogenesis of psoriasis.

In addition, recent studies indicate that cutaneous and systemic high-level expression of proinflammatory cytokines, such as IL-1β, IL-6, IL-12, IL-18, and interferon-γ (IFN-γ), are typically detected in psoriasis patients [12,13]. Notably, IL-18, which belongs to the IL-1 superfamily, is a representative proinflammatory cytokine that is highly expressed in psoriasis. IL-18, which has been demonstrated as playing an important role in cutaneous inflammation, is produced by monocytes, dendritic cells, epithelial cells, and keratinocytes. Recent studies have shown that the expression of IL-18 and its receptor (IL-18R) in psoriatic lesional skin is higher than the levels found in normal skin [14,15,16]. Additionally, plasma IL-18 concentration is significantly related to the severity of psoriasis [17,18]. In our previous studies, we found that erythroid differentiation regulator 1 (ERDR1) is negatively regulated by IL-18 and has an anti-cancer effect on murine melanoma through the inhibition of migration and invasive activity, in contrast to IL-18 [19]. ERDR1 was first observed in mouse leukemia cell lines and is considered a stress-related survival factor under stressful conditions [20,21]. ERDR1 is also expressed in normal human skin cells. In human keratinocytes, ERDR1 acts as a proapoptotic factor by enhancing ultraviolet B (UVB)-induced apoptosis, suggesting that ERDR1 may be a useful therapeutic target for psoriasis, which is characterized by a low level of apoptosis in hyper-proliferating keratinocytes [22].

In the present study, we evaluated the therapeutic effect of ERDR1 in psoriasis-like skin inflammation stimulated by imiquimod treatment. Recombinant ERDR1 significantly reduced the symptoms of psoriasis-like skin inflammation, including redness and scales in psoriatic lesional skin, and decreased the expression of inflammatory cytokines, especially those regulated by Th17 cells. In addition, downregulation of CCL20 and CCR6 by rERDR1 administration was also detected, implying that ERDR1 alleviates the psoriasis-like skin inflammation through the regulation of Th17 cell distribution in lesional skin. In conclusion, this study suggests that ERDR1 may be used as a novel strategy for the treatment of psoriasis.

## 2. Results

### 2.1. Recombinant Erythroid Differentiation Regulator (ERDR1) Improves the Symptoms of Psoriasis-Like Skin Inflammation in Vivo

**Figure 1:**
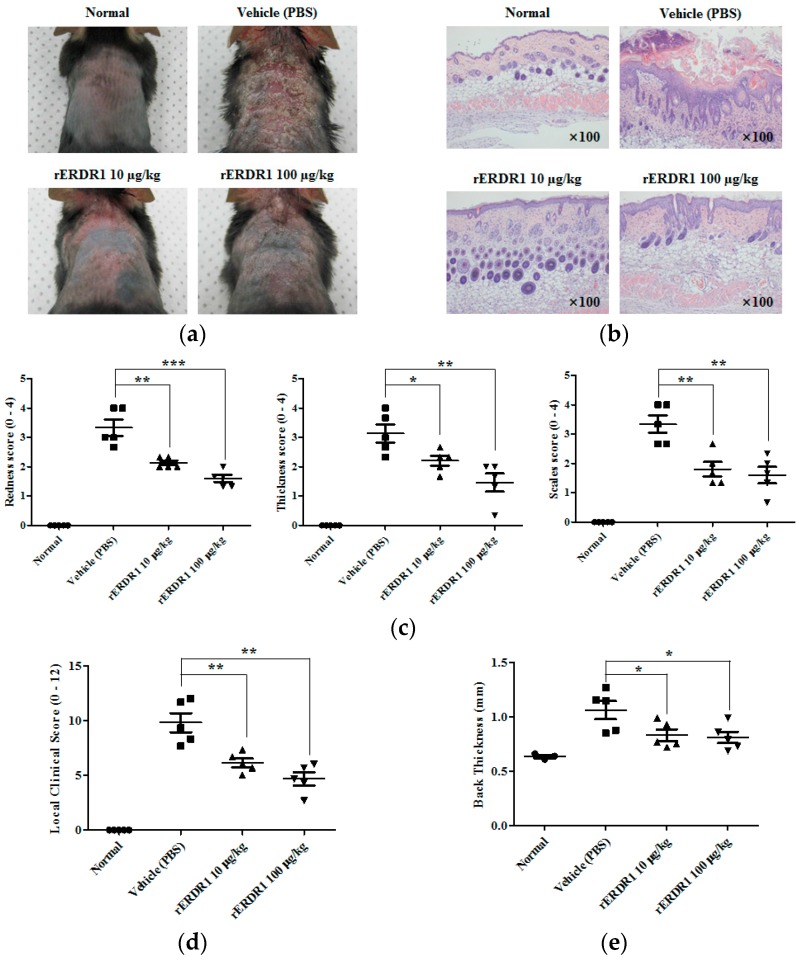
Recombinant erythroid differentiation regulator 1 (ERDR1) exerts therapeutic effects on psoriasis-like skin inflammation. (**a**) Photos showing redness and scales of psoriatic lesional skin induced by topical application of Aldara cream (5% imiquimod) on the shaved back skin of female 6-week-old C57BL/6J mice treated by 10 or 100 μg/kg intraperitoneally injected recombinant ERDR1 (rERDR1) versus PBS vehicle control. Five mice were used for each group, experiments were repeated independently three times, and data shown represent one mouse from each group; (**b**) H&E staining of the lesional skin shows decreased inflammatory infiltration, acanthosis, parakeratosis, and severe desquamation in rERDR1-injected mice compared to controls (×100 magnification); (**c**) Severities of redness, thickness, and scales of psoriasis-like skin lesions were evaluated from 0 to 4 (0, none; 1, mild; 2, moderate; 3, severe; and 4, very severe). Scoring was based on the severity of redness, thickness, and scales. Data are reported as mean ± standard deviation (SD). All values were analyzed using an unpaired Student’s *t*-test. * *p* < 0.05, ** *p* < 0.01, *** *p* < 0.0005; (**d**) The overall local clinical score was totaled from the sum of each category, including redness, thickness, and scales. Data are reported as mean ± SD. All values were analyzed using an unpaired Student’s *t*-test. ** *p* < 0.01; (**e**) Back skin thickness of the rERDR1-injected group *vs.* vehicle (PBS)-injected group, as measured using a thickness gauge. Data are reported as mean ± SD. All values were analyzed using an unpaired Student’s *t*-test. * *p* < 0.05. Data shown represent one of three independent experiments.

To investigate the therapeutic effect of ERDR1 on psoriasis, Aldara cream containing 5% imiquimod was topically applied to the shaved back skin of C57BL/6J mice to induce a psoriasis-like skin inflammation, and then 10 or 100 µg/kg of recombinant ERDR1 (rERDR1) was administered by intraperitoneal (i.p.) injection. PBS was used as a vehicle control. Figure 1a shows the external symptoms of psoriasis on the back skin of C57BL/6J mice. ERDR1 significantly improved these external symptoms, including redness and scales, in psoriatic lesional skin compared with the vehicle (PBS)-injected group. Histological analysis by H&E staining also showed that rERDR1 administration significantly decreased the inflammatory infiltration, acanthosis, parakeratosis, and severe desquamation (Figure 1b). Redness, thickness, and scales were scored from 0 to 4 to determine the severity of psoriasis (Figure 1c). The overall score was evaluated by the sum of each category. As shown in Figure 1d,e, the overall score and back thickness were considerably decreased by rERDR1 administration, whereas the scores were increased in the vehicle-injected group. Taken together, these data suggest that ERDR1 has therapeutic effects on psoriasis.

### 2.2. Recombinant ERDR1 Reduces the Expression of Biomarkers of Psoriasis

**Figure 2:**
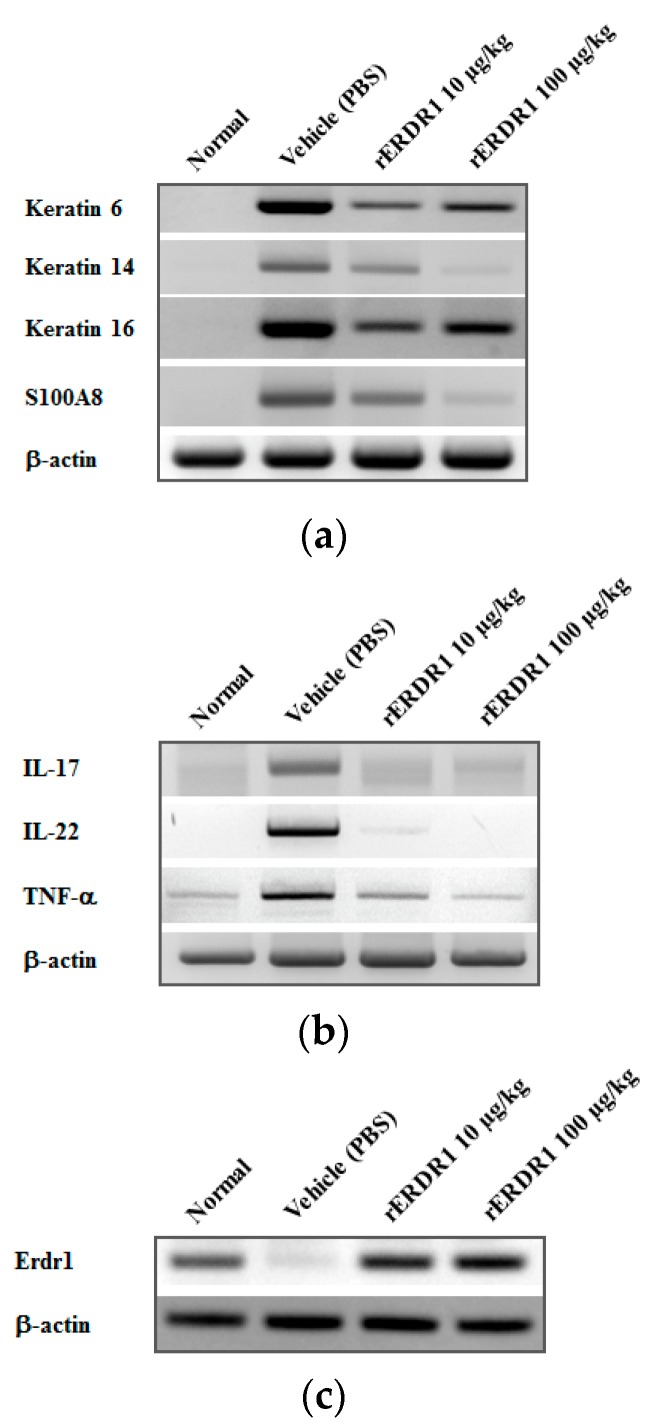
Expression of various biomarkers for psoriasis is regulated by rERDR1 administration. (**a**) Expression of hyper-proliferating marker, keratin 6, keratin 14, keratin 16, and antimicrobial peptide, S100A8, was detected by RT-PCR. The *Krt1*, *Krt14*, *Krt16* and *S100a8* mRNAs increased by imiquimod treatment were significantly decreased by rERDR1 administration; (**b**) Transcription of effector cytokines IL-17 and IL-22 that are produced by Th17 cells and act in psoriasis pathogenesis, as well as the inflammatory cytokine TNF-α, were detected in psoriatic lesional skin tissues. rERDR1 reduced mRNA expression of *IL-17*, *IL-22*, and *TNF-α*; (**c**) *Erdr1* mRNA expression was detected in psoriatic lesional skin tissues. *Erdr1* mRNA was significantly decreased following imiquimod treatment, and levels increased following rERDR1 administration. Data shown represent one of three independent experiments.

To examine the expression of psoriasis-related genes and inflammatory markers, RNA was extracted from skin tissues. As shown in Figure 2a, the mRNA expression of *Krt6*, *Krt14*, and *Krt16* which are associated with hyper-proliferation of keratinocytes, and of antimicrobial peptide *S100a8*, which is correlated with psoriasis severity, were significantly increased by imiquimod treatment in the vehicle (PBS)-injected group. However, in the rERDR1-injected group, the expression of *Krt6*, *Krt14*, *Krt16* and *S100a8* was significantly decreased, demonstrating that rERDR1 inhibits the induction of psoriasis-like skin inflammation by imiquimod. Additionally, inflammatory markers for psoriasis were also tested to confirm the therapeutic effects of rERDR1 administration. In order to test the effect of rERDR1 on various inflammatory cytokines including Th17 cytokines, gene expression was investigated in RNA extracts from lesional skin tissues. Figure 2b shows that imiquimod treatment induced *IL-17*, *IL-22*, and *TNF-α* in the vehicle (PBS)-injected group. However, the expression of *IL-17*, *IL-22*, and *TNF-α* in lesional skin was significantly reduced by rERDR1 administration. These data suggest that Th17 cell distribution may be reduced in the rERDR1-injected group compared with the vehicle (PBS)-injected group, implying that rERDR1 may control Th17 chemotaxis.

In addition, endogenous Erdr1 was examined in order to determine whether *Erdr1* mRNA expression is recovered as a result of treatment of psoriasis. As shown in Figure 2c, *Erdr1* mRNA expression was significantly decreased in imiquimod-induced psoriatic lesional skin. The decreased expression of *Erdr1* was considerably recovered by rERDR1 administration, suggesting the critical role of ERDR1 in psoriasis.

### 2.3. ERDR1 Inhibits Th17 Distribution in Psoriatic Lesional Skin through Regulation of CCL20 Expression

**Figure 3:**
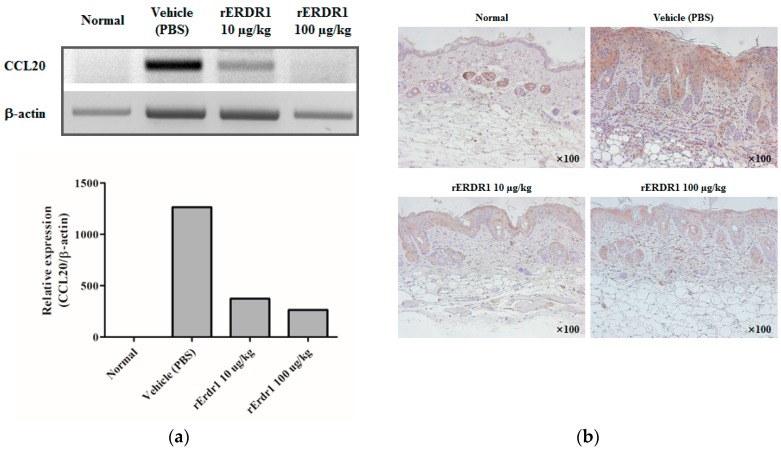
Recombinant ERDR1 inhibits CCL20 expression, resulting in decreased CCR6+ cells in psoriatic lesional skin. (**a**) Expression of CCL20, a chemokine for the trafficking of Th17 cells, was detected using RT-PCR. The increased *CCL20* mRNA expression by imiquimod treatment was markedly reduced by rERDR1 administration; (**b**) To detect CCR6 expression in the skin tissues, immunohistochemistry was performed with a polyclonal rat anti-mouse CCR6 antibody. Staining results were visualized under a microscope (original magnification; ×100). Data shows one of three independent experiments.

Recent study determines that CCR6+ Th17 cells have a critical role in psoriasis pathogenesis. IL-17 and IL-22, which are produced by Th17 cells, induce keratinocyte proliferation and inflammatory responses as part of the pathogenesis of psoriasis [7]. The CCR6+ Th17 cells migrate towards areas where the concentration of CCL20 is high. To test the involvement of rERDR1 in the CCL20-CCR6 chemotaxis system, mRNA expression of *CCL20* in lesional skin tissues was detected using RT-PCR. Figure 3a shows that *CCL20* expression was significantly decreased by rERDR1 administration, suggesting that rERDR1 can regulate the chemotaxis of CCR6-expressing cells. Next, CCR6 expression in lesional skin tissues was tested by immunohistochemistry using an anti-CCR6 antibody. In accordance with the decreased expression of ligand CCL20, its cognate receptor CCR6 was also downregulated in psoriatic lesional skin in the rERDR1-injected group (Figure 3b). As shown in Figure 3b, CCR6+ cells were abundant in psoriatic lesional skin following imiquimod treatment; however, their number was dramatically decreased by rERDR1 administration, showing that rERDR1 inhibits chemotaxis of CCR6+ Th17 cells via decreasing CCL20 expression. Taken together, these data suggest that ERDR1 regulates Th17 cell migration towards psoriatic lesional skin through the inhibition of the CCL20/CCR6 axis, resulting in the attenuation of psoriasis-like skin inflammation.

## 3. Discussion

Psoriasis is a common chronic skin disease that is accompanied by chronic inflammation. Immune cells and stressed keratinocytes secrete various proinflammatory cytokines to stimulate a variety of immune and resident skin cells, including keratinocytes. As a result, keratinocytes show abnormal hyper-proliferation and disturbed differentiation in the psoriatic lesional skin [3,23]. Various proinflammatory cytokines, including IL-18, IL-17, IL-12, and TNF-α, which are elevated in psoriatic lesional skin and have critical roles in the pathogenesis of psoriasis, have been identified and targeted for psoriasis treatment. Biological drugs, especially cytokine-directed therapies, have been developed to inhibit the functions of proinflammatory cytokines or to suppress the functions of immune cells, such as T cells [5,24,25]. A representative proinflammatory cytokine, IL-18, has been classified as a biomarker for the activity of psoriasis, in addition to other cytokines including transforming growth factor beta 1 (TGF-β1), tissue inhibitor of metalloproteinase 1 (TIMP-1), and matrix metalloproteinase 1 (MMP-1). In addition, serum IL-18 concentration and cutaneous IL-18 expression in the skin are significantly elevated in psoriasis patients compared with normal healthy donors, and there is a positive correlation between serum IL-18 expression and Psoriasis Area Severity Index (PASI) [17,26]. Therefore, suppression of the functions and expression of IL-18 is critical for the treatment of psoriasis.

In our previous studies, we determined that ERDR1 was elevated in a melanoma cell line, B16F10/IL-18 antisense, transfected with an antisense RNA against IL-18, indicating a negative correlation between ERDR1 and IL-18 expression. In addition to mouse melanoma, we also confirmed that ERDR1 expression was increased by IL-18 siRNA transfection in a human gastric cancer cell line [19,27]. Based on the negative correlation between ERDR1 and IL-18, we hypothesized that ERDR1 may exert anti-inflammatory effects, opposing the proinflammatory effect of IL-18. Particularly, ERDR1 plays an important role in cancer progression due to its suppression of tumor cell migration and metastasis in melanoma and gastric cancer, while IL-18 enhances these effects in these tumor types [19,27,28,29]. These studies show that ERDR1 and IL-18 have opposite effects. In addition, we previously reported that ERDR1 exerts a proapoptotic effect in the human keratinocyte cell line, HaCaT, suggesting that ERDR1 may be a useful therapeutic target for psoriasis, which is characterized by low apoptosis in hyper-proliferating keratinocytes [22]. A recent study also reported the therapeutic effects of ERDR1 on a rosacea-like mouse model through the inhibition of angiogenesis and inflammation, suggesting the crucial role of ERDR1 as an anti-angiogenic and anti-inflammatory factor [30]. In this study, Figure 2 shows that S100A8 was significantly decreased by ERDR1 treatment. It has been reported that S100A8 induces angiogenesis in psoriatic skin as well as increases keratinocyte proliferation [31]. Also, a recent study showed that S100A8 plays a key role in HIF-1α-induced angiogenesis, suggesting a proangiogenic role for S100A8 [32]. Therefore, ERDR1-regulated S100A8 may be involved in angiogenesis in the psoriasis mouse model. The detailed mechanisms will be investigated in our future studies.

As shown in Figure 1 and Figure 2, ERDR1 exerts a significant therapeutic effect on psoriasis-like skin inflammation induced by imiquimod treatment. Recombinant ERDR1 significantly alleviates histological symptoms, including inflammatory infiltration, acanthosis, parakeratosis, and severe desquamation, as well as external symptoms, such as redness, scales, and skin thickness. Various biomarkers for psoriasis were also decreased in the rERDR1-injected group along with the attenuation of psoriasis-like skin inflammation. In particular, Th17-related cytokines IL-17 and IL-22 were dramatically decreased by rERDR1 administration in psoriatic lesional skin, suggesting Th17 cell distribution in the lesional skin was reduced. These cytokines are predominantly produced by the Th17 cells, and activate keratinocytes, resulting in the hyper-proliferation of the epidermis and disruption of skin barrier functions [33,34]. IL-17 enhances the expression of proinflammatory cytokines (IL-1β and IL-6) and antimicrobial peptides (S100A7, S100A8, and S100A9) [35]. Additionally, IL-17 stimulates keratinocytes to produce various chemokines, such as CCL20, CXCL1, CXCL3, CXCL5, CXCL6, CXCL8 (IL-8), and β-defensin 2 [36]. The increased CCL20 in psoriatic lesional skin recruits CCR6-expressing cells, including Th17 cells and myeloid dendritic cells, contributing to the chronic inflammatory responses in psoriatic lesional skin. Thus, the induction of CCL20 has been suggested to play a crucial role in maintaining elevated Th17 cells in psoriatic lesional skin. In this study, ERDR1 regulates CCR6+ Th17 recruitment to psoriatic lesional skin via the downregulation of CCL20 expression as shown in Figure 3. Recent studies have determined that Th17 cells are highly associated with psoriasis along with other inflammatory diseases [7,10,37]. Based on increasing evidence for the importance of the CCR6+ Th17 cell population in psoriasis, we suggest that a decreased distribution of CCR6+ Th17 cells in psoriatic lesional skin via the regulation of CCL20 may be a critical mechanism for ERDR1-regulated psoriasis. In addition, it is well known that CCL20 production is increased by Th17 cytokines, such as IL-17 and IL-22 [38,39]. In this study, we confirmed that ERDR1 downregulates Th17 cytokines IL-17 and IL-22. This suggests that ERDR1 could inhibit CCL20 expression through the downregulation of IL-17 and IL-22. Also, recent study suggests an IL-22/STAT3/CCL20 signal cascade in cutaneous T-cell lymphoma [40]. The phosphorylated STAT3 binds to the CCL20 promoter, resulting in increased CCL20 expression [41]. Indeed, our recent study shows that ERDR1 inhibits STAT3 activity [42]. Therefore, we suggest that STAT3 may be involved in part of the signaling mechanism for CCL20 regulation by ERDR1. More detailed mechanisms will be pursued in future studies.

In addition to IL-17, IL-22 also plays an important role in the pathogenesis of psoriasis. Although IL-22 belongs to the IL-10 family, the former shows proinflammatory properties, in contrast to IL-10 [43]. IL-22 is also highly expressed in psoriatic lesional skin and activates keratinocytes to induce the production of antimicrobial peptides and the chemokine CXCL5. IL-22 stimulates the hyperplasia of keratinocytes though the inhibition of keratinocyte differentiation-related factors, such as filaggrin and keratin 10 [44], and enhances keratinocyte proliferation, resulting in acanthosis and pankeratosis [45]. Both IL-17 and IL-22 levels are elevated in the serum of psoriasis patients, and there is a positive correlation between serum levels of these cytokines and disease severity [46,47]. Therefore, the regulation of Th17 cell distribution has been suggested as a useful therapeutic target for the treatment of psoriasis.

## 4. Materials and Methods

### 4.1. Psoriasis Mouse Model

Female six-week-old C57BL/6J mice were purchased (Japan SLC Inc., Shizuoka, Japan) and maintained under conventional conditions. Psoriasis-like skin inflammation was induced on the shaved back skin of C57BL/6J mice by the topical application of Aldara cream containing 5% imiquimod (3M Pharmaceuticals, Northridge, CA, USA). The topical application was repeated daily for a week until the appearance of psoriasis-like skin inflammation. To examine the therapeutic effect of recombinant ERDR1 (rERDR1) on psoriasis-like skin inflammation, rERDR1 was injected intraperitoneally once a day for 7 consecutive days. Phosphate buffered saline (PBS) was injected as a vehicle control, while 10 or 100 µg/kg rERDR1 was injected to assess the effects of rERDR1 on the psoriasis-like skin inflammation. Each group was composed of five mice. The experiments were performed as three independent experiments. All experimental procedures with mice were approved by the Institutional Animal Care and Use Committee of Sookmyung Women’s University (Permission date: 18 June 2013; SM-IACUC-2013-0401-002).

### 4.2. Evaluation of Psoriasis-Like Skin Inflammation

Disease severity of psoriasis-like skin lesions was evaluated from 0 to 4 (0, none; 1, mild; 2, moderate; 3, severe; and 4, very severe). Scoring was based on the severity of redness, thickness, and scaling. The overall local clinical score was totaled from the sum of each category. Scores were measured by three researchers who were blinded to the grouping of the animals. To confirm the disease severity, back skin thickness was measured using a thickness gauge.

### 4.3. RNA Isolation, cDNA Synthesis, and RT-PCR

RNA extraction from psoriatic lesional skin tissues was performed using TRIzol reagent (Sigma-Aldrich, St. Louis, MO, USA). Reverse transcription was performed using 1 µg of RNA as a template with Moloney-Murine Leukemia Virus (M-MLV) reverse transcriptase, M-MLV 5× reaction buffer, RNase inhibitor (Promega Corp., Madison, WI, USA), 10 pmol Oligo (dT) primers, and 10 mM dNTPs (Bioneer, Daejeon, Korea) at 37 °C for 1 h. Synthesized complementary DNA (cDNA) was incubated with the primer sets for keratin 6 (*Krt6*), keratin 14 (*Krt14*), keratin 16 (*Krt16*), *S1008a*, *IL-17*, *IL-22*, *TNF-α*, *CCL20*, and β-actin. The cycling involved the steps of denaturation (95 °C, 30 s), annealing (55–60 °C, 30 s) and extension (72 °C, 30 s), with a final extension at 72 °C for 10 min.

### 4.4. Immunohistochemistry Analyses

For the histological evaluation, skin tissues were fixed in 4% paraformaldehyde overnight. Fixed skin tissues were embedded in paraffin, and then 8-µm sections were prepared. Sections were stained with hematoxylin and eosin (H&E) for visualization of the nucleus and cytoplasm. To detect CCR6 expression in the skin tissues, antibody against CCR6 was used (R & D Systems, Minneapolis, MN, USA). Briefly, the sections were blocked with blocking solution containing 5% goat serum, and incubated with monoclonal rat anti-mouse CCR6 antibody (1:100 dilution) overnight at 4 °C. After washing the sections, HRP-conjugated goat anti-rat IgG as a secondary antibody was added to the sections, and then 3,3’-diaminobenzidine (DAB; Invitrogen Life Technologies, Carlsbad, CA, USA) was used as a substrate to detect expression of the target protein. Staining results were visualized under a microscope.

### 4.5. Statistics

All values were analyzed with an unpaired Student’s *t*-test. Statistical analyses were performed using GraphPad Prism 5 (GraphPad Software, La Jolla, CA, USA). *p* values < 0.05 were considered statistically significant in a two-tailed *t*-test.

## 5. Conclusion

In conclusion, this study provides the first evidence for the therapeutic effect of ERDR1 on psoriasis. Recombinant ERDR1 injection alleviates psoriasis-like skin inflammation induced by imiquimod treatment through the inhibition of Th17 recruitment to psoriatic lesional skin. Therefore, we suggest that ERDR1 may be a potential therapeutic target for psoriasis.
